# Current Status and Future Perspectives of Lactate Dehydrogenase Detection and Medical Implications: A Review

**DOI:** 10.3390/bios12121145

**Published:** 2022-12-07

**Authors:** Yangzhe Zhou, Min Qi, Minghui Yang

**Affiliations:** 1 Department of Plastic Surgery, Xiangya Hospital, Central South University, Changsha 410008, China; 2 College of Chemistry and Chemical Engineering, Central South University, Changsha 410083, China

**Keywords:** lactate dehydrogenase, quantum dots, fluorescence, nicotinamide adenine dinucleotide, tumor

## Abstract

The demand for glucose uptake and the accompanying enhanced glycolytic energy metabolism is one of the most important features of cancer cells. Unlike the aerobic metabolic pathway in normal cells, the large amount of pyruvate produced by the dramatic increase of glycolysis in cancer cells needs to be converted to lactate in the cytoplasm, which cannot be done without a large amount of lactate dehydrogenase (LDH). This explains why elevated serum LDH concentrations are usually seen in cancer patient populations. LDH not only correlates with clinical prognostic survival indicators, but also guides subsequent drug therapy. Besides their role in cancers, LDH is also a biomarker for malaria and other diseases. Therefore, it is urgent to develop methods for sensitive and convenient LDH detection. Here, this review systematically summarizes the clinical impact of lactate dehydrogenase detection and principles for LDH detection. The advantages as well as limitations of different detection methods and the future trends for LDH detection were also discussed.

## 1. Introduction

Lactate dehydrogenase (LDH) is one of the most common enzymes in nature with an Enzyme Commission Number (EC)^C^ of 1. 1. 1. 27 [1,2]. Structurally, LDH is a tetrameric enzyme which mainly consists of two subunits, LDHA and LDHB. The two subunits are encoded by two independent genes, LDHA and LDHB, respectively [1,3]. LDHA and LDHB are expressed in skeletal muscle and cardiac muscle, respectively [4]. These two subunits can also be combined into five different forms to form homomers or heterotetramers in the human body, including: LDH−1, LDH−2, LDH−3, LDH−4 and LDH−5 [5]. The most critical reaction in which LDH is involved in the body is the conversion of lactic acid and oxidized nicotinamide adenine dinucleotide (NAD) to pyruvate and reduced nicotinamide adenine dinucleotide (NADH), respectively [6,7,8]. It is worth noting that LDHA has a higher affinity for pyruvate, which preferentially converts pyruvate to lactate while oxidizing NADH to NAD. Conversely, LDHB has a higher affinity for lactate, which preferentially convert lactate to pyruvate while reducing NAD to NADH [6,9,10,11]. This reversible reaction is sensitive to different conditions and was also applied in our previous LDH assay experiments [12].

**Figure 1:**
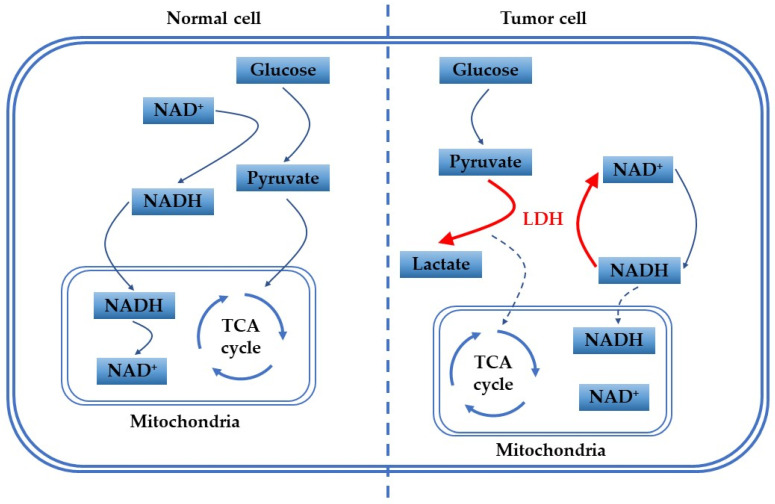
Difference of glucose metabolism between normal and tumor cells in vivo.

It is known that the pyruvate produced in normal cells is mainly delivered to the inner mitochondrial membrane, where it is oxidized by pyruvate dehydrogenase complex to acetyl coenzyme A (CoA). Then CoA then enters the tricarboxylic acid cycle, where it is oxidatively phosphorylated (OXPHOS) for the efficient production of ATP for energy supply [11,13,14,15]. However, tumor cells are out of the ordinary. In the early 20th century, the German biochemist Otto Warburg explained the phenomenon that tumor cells produce large amounts of lactic acid even in the presence of sufficient oxygen. This is now known as the Warburg effect [11,13,16,17,18,19,20]. The lactate produced here is due to the dramatic increase of glycolysis rate in tumor cells, which occurs as the produced pyruvate is converted to lactate by the action of LDH without entering the mitochondria to be metabolized by oxidation [1,6,11,21]. This explains why LDH is usually elevated in cancer patients. Figure 1 illustrates the difference between the metabolism of normal cells and tumor cells in vivo. According to recent reports, dual inhibition of OXPHOS and glycolysis can disrupt the plasticity of tumor cells, curbing their energy supply and generating effective therapeutic options [22,23]. For example, Jennifer R. Molina et al. discovered IACS−010759, a substance that strongly inhibits tumor growth and induces apoptosis, which can be used to treat acute myeloid leukemia and brain cancer by inhibiting OXPHOS [24]. Other researchers also have found that by inhibiting OXPHOS in this pathway can be used to treat cancers such as pancreatic cancer and triple negative breast cancer [25,26]. In addition, Daan F. Boreel et al. found that targeting OXPHOS could be used to improve the efficacy of radiation and immunotherapy treatments, which may be a new approach for future tumor treatment [27].

In healthy human serum, LDH concentration should not be higher than 200 U/L. This figure is the standard in routine clinical blood sampling for LDH testing. When elevated LDH levels are found, we are alerted to the possible presence of a tumor or residual tumor remaining after a tumor resection surgery. This is because LDH levels will drop significantly within one to two weeks after complete tumor removal [28]. At the same time, low levels of LDH are associated with good prognosis and complete tumor resection [29,30,31]. Currently, LDH has become a consensus as a biomarker for tumors, and plays a very important role as a prognostic survival indicator and guides drug treatment for cancer patients. For instance, numerous studies have reported LDH as a prognostic biomarker for colorectal cancer [32,33], lung cancer [34,35], melanoma [36,37], renal cell cancer [38,39] and hepatocellular carcinoma [40,41]. Many studies have also shown that LDH is also a biomarker for malaria [42,43,44]. Moreover, LDH activity is an important clinical guide to the choice of chemotherapy and helps to identify whether a pleural effusion is benign or malignant [45,46]. In addition, Sander Kelderman’s team found that ipilimumab was less effective when serum LDH levels were higher than twice the normal value, and S. Diem et al. elucidated that LDH is predictive for melanoma patients receiving PD−1 therapy [47,48]. Since the outbreak of the novel coronavirus pneumonia in 2019 (COVID-19), it seriously endangers the survival and health of human beings. Some researchers have even discovered that elevated LDH is associated with an increased risk of severe COVID-19, which can be used as a survival indicator of poor prognosis [49,50,51,52,53,54,55,56,57,58,59].

**Figure 2:**
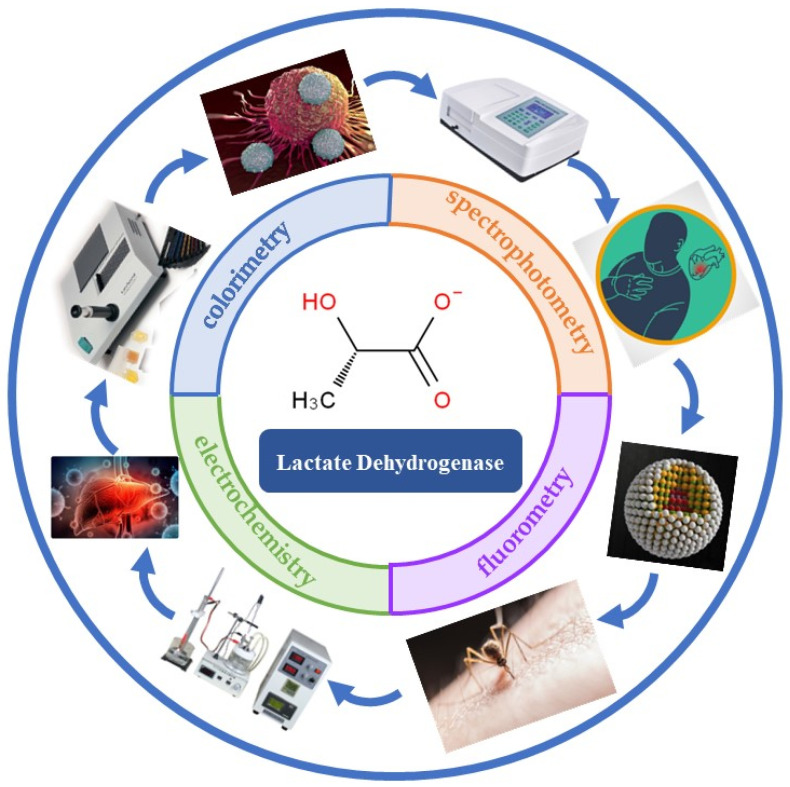
Lactate dehydrogenase applications in medical sectors.

In summary, LDH plays an important role in the medical field, especially as a biomarker for tumors. Figure 2 illustrates the applications of LDH in medicine. For these reasons, it is urgent to develop sensitive and convenient methods for LDH detection.

Many methods have been reported for the quantitative detection of LDH, including colorimetry [60,61,62,63,64,65,66,67,68,69], spectrophotometry [70,71,72,73,74], electrochemistry [75,76,77,78,79,80,81,82] and fluorometry [12,61,74,83,84,85,86,87]. This article reviews the various methods for LDH detection, discusses their advantages as well as limitations and looks forward to future trends.

## 2. Various Substances Involved in LDH Testing

The most important reaction in which LDH involved is the conversion of lactate and NAD to pyruvate and NADH. The reaction equation is as follows:

$$\mathrm{Lactate}\;+\;{\mathrm{NAD}}^{+}\overset{\;\mathrm{LDH}\;}{↔}\mathrm{Pyruvate}\;+\;\mathrm{NADH}\;+\;H^{+}$$

Unlike other methods that directly detect the analyte, the detection of LDH is usually achieved through the indirect measurement of the substances involved in the redox reactions in which LDH participates. The two most critical substances in this reaction are NADH and its oxidized form NAD. Both play a crucial role in biological systems [88,89,90]. NADH is an essential oxidation reducer and is primarily responsible for bringing electrons from the tricarboxylic acid cycle into the electron transport chain to generate energy in the form of ATP [91]. Its oxidized form, NAD, is the best receptor for the reducing equivalents generated by the oxidation of various substrates in the cell. NAD receives reducing equivalents from the citric acid cycle and glycolytic processes, which also play a key role in cellular energy metabolism [92,93]. An imbalance in the ratio of NADH to NAD leads to a disruption of energy production by these pathways, which in turn leads to dysregulation of cellular metabolism [94,95].

## 3. LDH Detection Methods

### 3.1. Colorimetric Method

**Figure 3:**
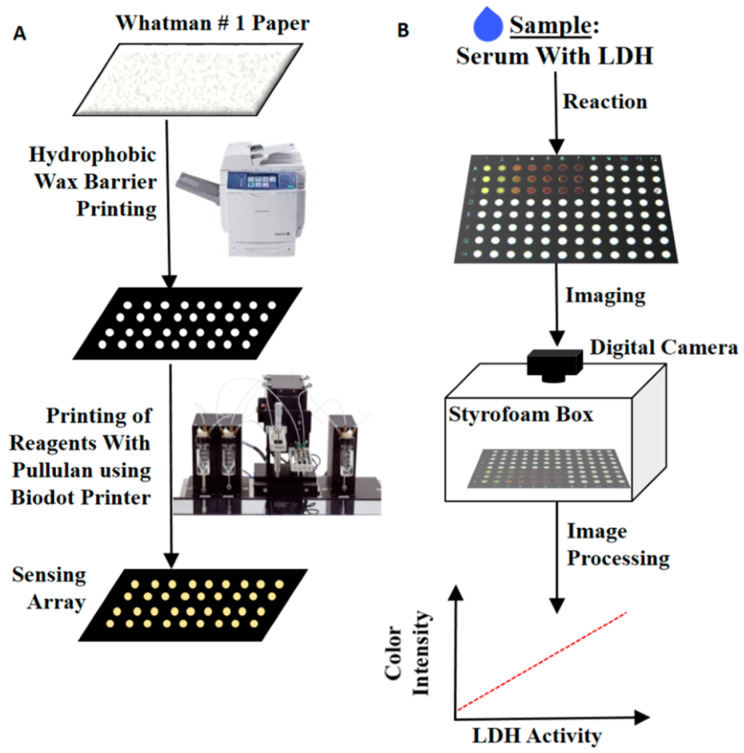
(**A**) Preparation of paper-based LDH sensors. (**B**) Assay for serum LDH [98]. Copyright 2015 American Chemical Society.

A colorimeter is a laboratory instrument that details the concentration represented by a color and is widely used for the routine measurement of substance concentrations because of advantages such as affordability, portability and direct visual observation of the results [64,96,97]. As shown in Figure 3, Kannan et al. developed a highly stable and mass-produced colorimetric biosensor for LDH detection, which can be operated in less than 5 min and has a detection limit as low as 13 U/L. The sensor was based on the principle of fixing Pullulan on paper. The serum containing LDH on the Pullulan-fixed paper is then added for colorimetric analysis. The sensor can be printed onto paper holes in a highly reproducible manner using an automated printing system, allowing the production of sensors in line with high-speed automated manufacturing. The color development after the addition of LDH can be seen with the naked eye and quantified with a digital camera and image processing software. The sensor uses an aggressively low volume of reagents, significantly reducing the costs associated with the assay. The paper wells can also be stored at room temperature for short periods of time (2–3 weeks) and in a refrigerator for at least five weeks. This eliminates the need for complex laboratory facilities, expensive reagent transport and storage and complicated sample handling. Therefore, the sensor can be used for rapid, inexpensive screening of large numbers of samples in resource-limited settings [98]. Moreover, Arias et al. reported a similar paper-based sensor which is based on a single-step magnetic immunoassay and can be performed in less than 20 min on an inexpensive and simple paper-based disposable device. The assay consists of a single incubation of the lysed whole blood sample with the reagent mixture for 5 min. The mixture is then pipetted directly into a single piece of paper-based equipment, manufactured using a low-cost process cuter. A detection limit of 0.39 U/L can be achieved by visual colorimetry [99]. In addition, Papaneophytou et al. indirectly detected LDH through the LDH-catalyzed production of NADH by reacting it with nitro blue tetrazolium (NBT) and phenazine methyl sulfate (PMS), resulting in a change in the color of the mixture followed by the formation of blue–purple beetles [69]. Furthermore, Halvorsen et al. reported an early prototype of a manually manufactured rapid paper-based point-of-care (POC) assay that required minute amounts of whole blood and used a smartphone camera to provide colorimetric LDH concentration measurements in less than 4 min. The POC analysis device involved lateral separation of whole blood into plasma on a set of filter papers, a colorimetric membrane using a dry chemical reaction on a filter membrane and analysis of the concentration using software on a smartphone. The ease of operation and smartphone-based readings make this POC platform particularly suitable for resource-limited environments. The smartphone provides real-time output of a colorimetric test that is completely self-contained, with a mobile application performing the data analysis. The smartphone can also be connected directly to an external computer. Mobile communication technology facilitates information management. Finally, POC systems are relatively inexpensive to manufacture and can be used as disposable device. These methods eliminate the need for complex laboratory facilities and complicated sample analyses, allowing for rapid, low-cost screening of large numbers of samples in resource-constrained medical settings [98,100]. However, the colorimetric method can only be applied to samples with relatively simple composition that are less susceptible to interference. Moreover, its relatively low sensitivity is obstacle to its wide application [60,96].

### 3.2. Spectrophotometric Method

The most commonly used spectrophotometry method is UV-spectrophotometry, which is easy to implement and often used for the detection of various substances [2,101]. NADH has one absorption peak at 260 nm and one at 340 nm, while NAD has only one absorption peak at 260 nm. This important property distinguishes the two, and is also the physical basis for measuring the metabolic rate in many metabolic tests [102]. Damaris et al. developed a facile spectroscopic assay for detection of LDH in saliva, where the assay is based on the interconversion of catalytic pyruvate and lactate in the presence of LDH, and the decrease in absorbance at 340 nm caused by NADH is proportional to the LDH activity in the sample [103]. In addition, Lee’s team reported a microfluidic microplate-based immunoassay, which requires only a small amount of antibody for faster detection of LDH compared to traditional ELISA. The entire body of this microplate consists of a 96-well plate that has an inlet for pipette injection, an outlet open towards the absorbent pad and a microfluidic channel between them. The microfluidic microplate is a spiral microfluidic channel with 1.5 times larger surface area and 50 times larger surface area-to-volume ratio compared to conventional ELISA plates. The detection limit is as low as 6.25 × 10^−3^ U/L [43]. However, the UV spectrum also will be easily affected by the external environment, such as the color of the sample [70]. 

### 3.3. Electrochemical Measurement

Electrochemical detection is based on changes of the electrical signal due to the substance to be measured. This method is popular because of its low cost, fast response and ease of miniaturization. There are a wide variety of electrochemical biosensors based on different electrochemical techniques, such as cyclic voltammetry (CV), differential pulse voltammetry (DPV), stripping voltammetry, alternating current voltammetry (ACV), polarimetry, square wave voltammetry (SWV) and linear scanning voltammetry (LSV) [104,105].

**Figure 4:**
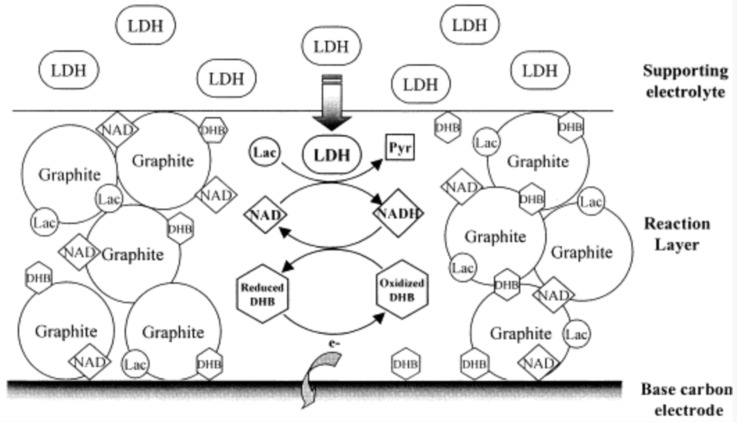
Response scheme for thick film LDH biosensors [106]. Copyright 2002 Elsevier.

As shown in Figure 4, Hong et al. fabricated an electrochemical sensor based on screen-printed electrodes for LDH detection. The reaction layer of the printed ink used for the working electrode consisted of graphite, electrodeposited 3,4−DHB, NAD and l−lactate, which were bound in a composite polymer binder. LDH, the target analyte, diffused from the supporting electrolyte into the reaction layer and reduced NAD to NADH. The generated NADH was then oxidized on the electrode surface, generating electrochemical current that is proportional to LDH activity. This method can detect LDH in the range of 50–500 U/L with a detection limit of 50 U/L, which is sufficient to meet the basic needs of the test [106]. In addition, Zhu et al. developed a novel detection platform combining microfluidics and electrochemical sensors arrays, which contains three parts: sample processing, detection and signal output. The processing analysis starts when the sample flows into the chip, uses lactic acid as the substrate. LDH catalyzes the reduction of NAD to NADH. Then, the concentration of LDH is assessed by electrochemical detection of NADH. It establishes a linear relationship in the range of 60–700 U/L with a detection limit of 25 U/L, which is much lower than the serum LDH concentration at the time of tumorigenesis [107]. Furthermore, researchers have tested a zwitterionic phenazine compound: 3−(1−methoxyphenazin−5−ium−5−yl)propane−1−sulfonate(mPPS), which acts as an electron mediator for the electrochemical oxidation of NADH. LDH was detected by the redox reaction of NAD to NADH with a detection limit as low as 0.5 U/L [108]. Gisela’s team developed a point-of-care (POC) device that includes a single-use microfluidic paper, double-sided, screen-printed carbon electrode (MP-dsSPCE). The POC requires an optimized single-step immunoassay performed using magnetic beads and an immunomodified signal amplifier that is carried primarily in MP-dsSPCE. The system is capable of performing LDH assays in less than 20 min with minimal user intervention, and can provide a detection limit of 50 U/L [109]. In addition, Xu et al. developed an immunosensor for electrochemical detection of LDH. Firstly, multi-walled carbon nanotubes are assembled with gold nanoparticles onto electrode surface, which increases the surface area of the electrode and improves the conductivity of the electrode. LDH antibodies are then modified onto the electrode surface for capturing LDH. When LDH is immobilized on the electrode surface, it catalyzes the formation of pyruvate and NADH in the substrate solution, which enhances the current signal. Conversely, when the LDH concentration is low, the signal is weakened so that a linear relationship between LDH concentration and current intensity is established. The detection range of this method is 0.55–275 U/L, and the detection limit is 0.21 U/L [110,111,112,113]. 

**Figure 5:**
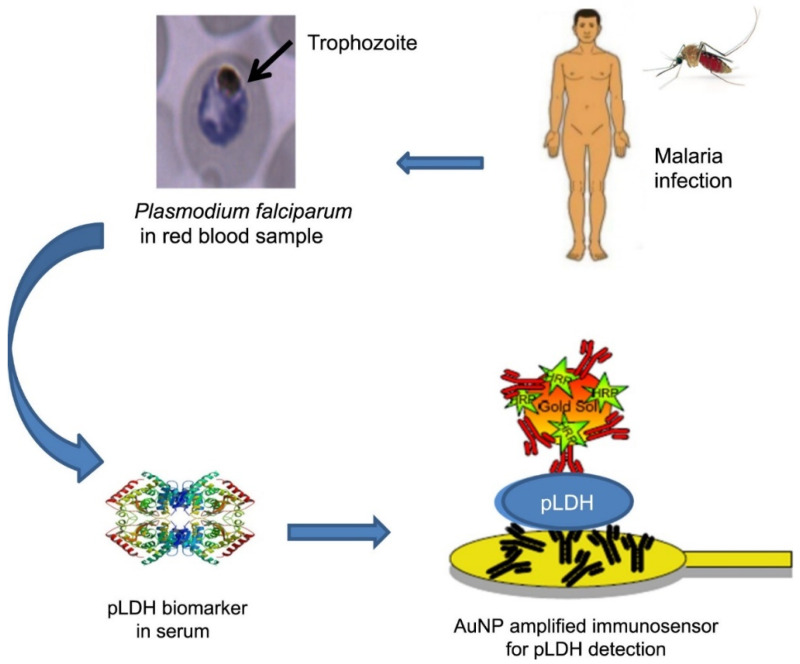
Schematic diagram of the process of LDH detection by immunosensor [115]. Copyright 2018 Elsevier.

Immunoassays have been widely used for the detection of LDH [114]. Figure 5 shows the work of Hemben et al., who developed an immunosensor using gold nanoparticles to enhance sensitivity. When humans are infected with malaria by mosquito bites, LDH rises rapidly in the blood. LDH is then detected by a gold nanoparticle-modified immunosensor during the active phase. In comparison with commercial kits, the sensor showed higher sensitivity and better reproducibility, allowing for immediate detection at low cost. The sensor is capable of detecting LDH in the range of 0–0.17 U/L with a detection limit as low as 0.45 U/L and can be used with a networked mobile device, which can greatly facilitate the detection process [115]. 

### 3.4. Fluorometric Measurement

When a fluorescent substance is irradiated by incident light of a certain wavelength (usually UV light), the substances absorb light energy, enters the excited state, and immediately de-excites and emits light longer than the wavelength of the incident light (usually in the visible wavelength). Many fluorescent substances cease to emit light once the incident light stops, and the luminescence disappears immediately [116]. Compared with the previous detection methods, fluorescence analysis has advantages of high sensitivity, high throughput, wide linearity range, making it a good choice for LDH detection [117,118].

**Figure 6:**
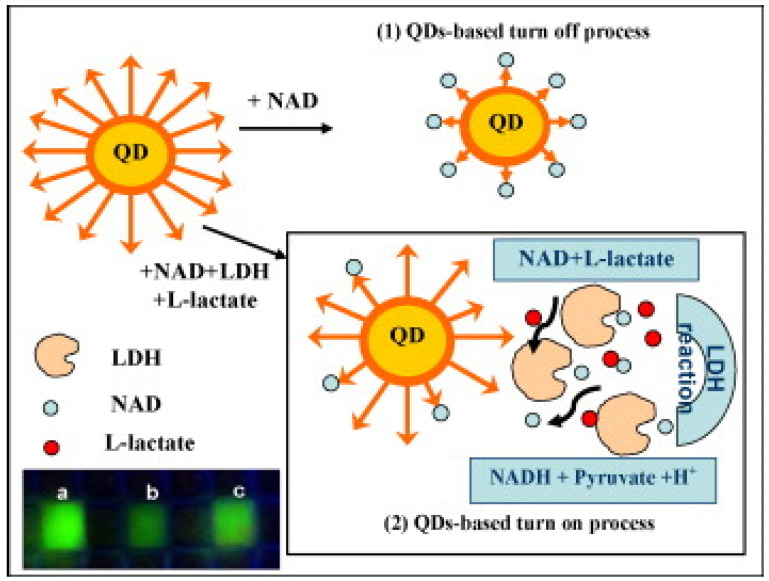
Schematic diagram of the principle of LDH determination, with the lower left panel showing the fluorescence image of the QDs in the absence (**a**) or presence (**b**) of NAD, and in the presence of LDH, NAD and LL (**c**) [86]. Copyright 2010 Elsevier.

**Figure 7:**
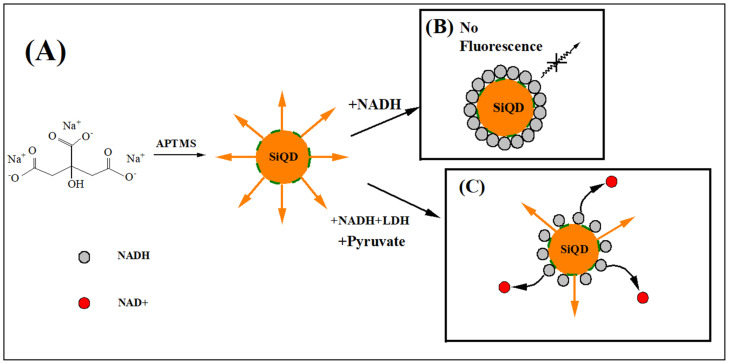
(**A**) The synthesis principle of SiQDs. (**B**) NADH quenches fluorescence of SiQDs. (**C**) Principle of LDH activity detection [12]. Copyright 2022 Elsevier.

**Figure 8:**
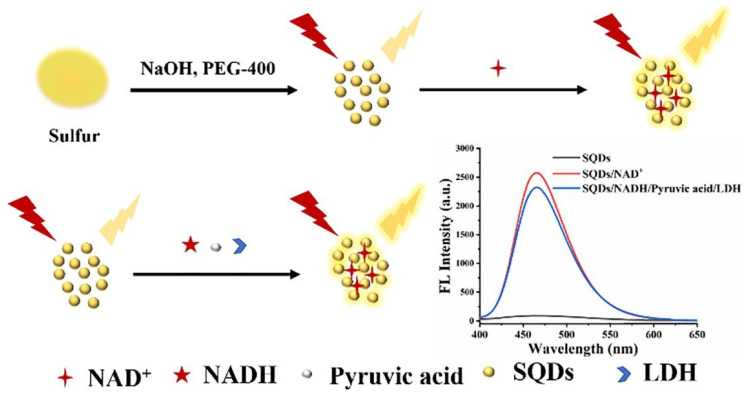
Schematic diagram of LDH detection by SQDs. Copyright 2022 Elsevier.

Back in 2010, Ren et al. reported the detection of LDH activity based on CdTe/CdS quantum dots. As shown in Figure 6, in this approach, the fluorescence of QDs was first quenched by NAD, caused by chemisorption or electrostatic diffusion of NAD onto the surface of QDs, which affecting the chemical bonds on QD surface. Then the fluorescence was gradually recovered with the addition of LDH, which was ascribed to the reduction of NAD to NADH catalyzed by LDH, thus consuming NAD and weakening the quenching effect of NAD on the fluorescence of QDs. The detection limit of this method was 75 U/L, and it has a good linearity in the range of 150–1500 U/L with a correlation coefficient of 0.996 [86]. One year later, they developed a CdTe QDs-based assay for LDH detection and successfully applied it to detect LDH in human serum samples, making up for the shortcomings of the previous work. The linear range of the assay is 250–6000 U/L, and the assay can be completed in 15 min [84]. Our team also developed silicon quantum dots (SiQDs) and sulfur quantum dots (SQDs) based assays for LDH detection, which have wide linear range for LDH detection. As shown in Figure 7, in the work on SiQDs, at an excitation wavelength of 350 nm, the synthesized SiQDs possess an emission peak at 450 nm. We found that no significant quenching effect was achieved when we mixed SiQDs with NAD. On the contrary, NADH has a significant quenching effect on SiQDs. The principle of fluorescence quenching is due to the diffusion of NADH onto the surface of SiQDs, leading to the electron transfer (ET) process on SiQDs. The detection limit is 970 U/L. To verify the feasibility of LDH detection by SiQDs, we conducted several control experiments. When NADH was added to the SiQDs solution, the fluorescence intensity of SiQDs was significantly reduced, indicating that NADH has a strong quenching effect on SiQDs. Then, the fluorescence intensity was restored after continuing to add LDH together with pyruvate to the mixed solution described above. However, when SiQDs were reacted with LDH alone, the fluorescence intensity did not change significantly. In addition, we also investigated the quenching of fluorescence intensity of SiQDs by NAD and the recovery of fluorescence with the addition of LDH. Although NAD can also quench the fluorescence of SiQDs, the quenching effect was not as significant as that of NADH. The fluorescence intensity was not recovered when LDH and pyruvate were added, indicating that LDH could not catalyze the conversion of NAD to NADH. Since LDH-catalyzed enzymatic reactions are reversible, we believe that the above phenomenon is attributed to the fact that it is much easier for LDH to catalyze pyruvate to lactate, while it is relatively difficult for LDH to catalyze lactate to pyruvate. Due to the presence of many interfering substances in human serum samples, the effect of various substances on the assay was also investigated to study the selectivity of the assay. The sensitivity of the method for LDH was much higher than that for other substances, indicating that the method has a high selectivity for the detection of LDH. As shown in Figure 8, in the experiments on SQDs, it was shown that NAD can increase the fluorescence intensity of SQDs, and its fluorescence intensity increased by about 30 times when 1 M of NAD was added. This is due to the fact that the emission wavelength of NAD is close to the excitation wavelength of SQDs, and the energy transfer between them enhances the fluorescence intensity of SQDs. A detection limit of 262.41 U/L can be achieved in the linear range of 0.5–40 × 10^3^ U/L [119]. Compared to CdTe and CdTe/CdS QDs, SiQDs and SQDs have unique advantages such as good biocompatibility, low toxicity, good water solubility and photostability [12,119]. 

Wu’s team used super-bright adenosine monophosphate (AMP)-covered gold nanoclusters (AuNCs@AMP) as a fluorescent probe to detect LDH. In contrast to the previous use of NAD or NADH as a quencher, they found that LDH could be used as a quencher for this probe. The mechanism of this quenching is due to the formation of Au-thiol complex by the free sulfhydryl groups in LDH in the microenvironment on the protein surface. When excited at 328 nm, AuNCs@AMP exhibited an intense fluorescence peak at 480 nm and their fluorescence emission was gradually quenched with time in the presence of 2.0 μM LDH. This resulted in an intensity loss of nearly 70% with a slight blue shift. The method was able to detect LDH linearly in the concentration range of 8–400 U/L with a detection limit of 0.8 U/L [120]. Building on their previous work, their team developed a quantitative assay for LDH based on Au−AgNCs@AMP. This time, LDH was instead used to enhance the fluorescence intensity of the probe, which could reach up to 5 times the initial intensity in the presence of 1.0 μM LDH. The mechanism suggests that the probe interacts with the structural domain of LDH near the active site and is driven by electrostatic interactions towards the free thiol group. The fluorescence enhancement was attributed to assembly-induced emission enhancement (AIEE) and hydrophobic transfer. Interestingly, they also introduced Al^3+^ in order to target specific LDH for detection. Al^3+^ can bind to CuNCs@GSH and AgNCs@GSH, forming aggregates through electrostatic interactions and producing strong emission enhancement effects. These results suggest that Al^3+^ are suitable promoters for improving the emission of nanoclusters and extending their application. Al^3+^ plays an important role in the specific detection of PvLDH by shielding the fluorescence response of Au−AgNCs@AMP to RLDH, PfLDH and HLDH, but maintains the response to PvLDH. Therefore, specific LDH species can be detected more specifically [83]. 

In addition, He et al. reported a fluorescence sensor based on CdSe quantum dots/ polycaprolactone (PCL) composite electrospun fluorescent porous fiber membranes, where CdSe QDs were uniformly distributed within the PCL fiber as fluorescent probes, resulting in fluorescence quenching due to electron transfer (ET) between NAD and CdSe QDs. In order to accelerate the diffusion of analytes inside the fiber and to improve sensitivity, a porogenic agent was introduced to produce a secondary porous structure in the fiber. Compared with the fluorescence quenching sensor, this luciferase sensor can effectively reduce the background signal, avoiding the false signal interference caused by other quenching agents in the actual sample, which improves the sensitivity and selectivity of the sensor. Each assay takes only 10 min and is linear in the range of 200–2400 U/L [121]. Moreover, Kenry et al. reported a new application of monolayer MoS_2_ nanosheets in the development of “catch-release” aptamer biomolecular sensors. Unlike chemisorption and electron transfer, the mechanism is as follows: the MoS_2_ nanosheets first quench the LDH aptamer solution with fluorescent properties, and then the target LDH protein induces the release of the aptamer from the nanosheet surface to restore fluorescence. The linearity range is 0–578.13 U/L and detection limit is 5.09 U/L [122]. 

Unlike conventional quantum dots, Jenie developed a Resazurin based fluorescent probe to detect LDH. Resazurin, known as Alamar Blue, has weak fluorescent properties. With the transfer of electrons, the heterocyclic N−oxide group in Resazurin loses oxygen upon reduction and forms the strongly fluorescent product—resorufin. The porous silicon with microcavity structure enhanced the fluorescence signal. This is because the microcavities are multilayers consisting of intervening spacer layers and alternating porosity. The microcavities are capable of enhancing the emission of fluorophores confined in the porous structure according to the Purcell effect. The fluorescence intensity measured when LDH was present on porous silicon microcavities was ten and fives times higher than that of monolayers and detuned microcavities, respectively. The linear range of the assay system is 0.16–6.5 × 10^3^ U/L and the detection limit is 80 U/L [123]. Dr. Minopoli presented a detection system based on plasma-enhanced fluorescence assay combining the plasma characteristics of AuNPs and a unique photochemical functionalization technique. The photochemical immobilization technique provides a fast and simple strategy to covalently tether antibodies to a gold surface, exposing fragment antigen binding sites to the surrounding environment. The assay is performed in a sandwich configuration where antibodies act as a capture bioreceptor and a fluorescently labeled aptamer binds to LDH from the top. AuNPs are used as fluorescent enhancers to improve the sensitivity of the assay [124]. Furthermore, Dr. Alpizar reported a paper-based fluorescent magnetic immunoassay that begins with a 5 min immunocapture in a test tube. The mixture is then transferred to the distal end of the paper device wash pad. When it is absorbed, 500 μL of PBS–T is added to the wash bath. This pushes the mixture into the magnetic particles’ concentration fraction, where the magnet retains the magnetic particles while the unbound reagents flow toward the end absorbent pad. Finally, 50 μL of QuantaRed is added to the bottom of the wash pad and the device is incubated in the dark for 5 min. Fluorescence detection was achieved using a homemade portable fluorometer. The assay platform is capable of providing a detection limit of 0.225 U/L over a linear range of 0.2–3.13 U/L [99]. 

According to relevant studies, the normal range of LDH in human serum is 100–300 U/L. When the LDH level exceeds 1000 U/L, it suggests the possibility of related diseases or tumors. This indicates that the linear range of these current test are sufficient. Table 1 summaries the performance of recently reported assays for LDH.

## 4. Future Prospects

LDH plays an important role as a biomarker for different diseases such as cancers and malaria. In this review, we summarized various assays for LDH and discussed their advantages and limitations. Although different methods have been reported for LDH analysis, which generally meet most basic detection needs, further efforts are still needed to improve the performance for LDH detection for early screening of diseases. In the future, the main challenges we need to overcome are improvement of sensitivity, reduction of the cost and development of point-of-care testing (POCT). For example, by utilizing properties of nanomaterials, we can synthetize probes with dual fluorescence emissions to develop ratiometric assays for LDH. By using one emission peak as a built-in correction and the other emission as a signal, ratiometric probes can eliminate variability arising from environmental interference, leading to improved sensitivity and accuracy. Near-infrared fluorescence probes can also be developed for in vivo study and cellular imaging of LDH activity. Near-infrared fluorescence probes have been extensively used in biological detection and imaging due to their attractive properties, including large anti-Stokes shifts, good photostability, minimized autofluorescence and especially deep tissue penetration in biological samples. Based on electrochemistry, similar to a glucose testing strip, POCT for LDH can also be developed so that people can monitor LDH activity routine at home. Moreover, although many methods have been recently reported for LDH detection, the commercialization of these methods still needs further efforts, such as improving the reproducibility, selectivity as well as precision of the methods. More clinical samples need to be tested by the methods. More clinical data revealing the relationship of LDH with other diseases are required to find wider applications of LDH as clinical biomarkers for diseases diagnosis and therapeutic treatment. 

## Figures and Tables

**Table 1:** Performance of recently reported LDH detection methods.

Analytical Method	Materials	Detection Limit (U L^−1^)	Linear Range (U L^−1^)	Sensor Response Time (min)	Correlation Coefficient	Reference	Real Sample Test
Fluorescence	CdTe QDs	**/**	250–6000	15	0.996	[84]	Serum
Fluorescence	CdTe/CdS QDs	75	150–1500	20	0.996	[86]	**/**
Fluorescence	AuNCs@AMP	0.8	8.0–400	30	0.996	[120]	Serum
Fluorescence	Au−AgNCs@AMP	0.93	92.5–925	10	0.997	[83]	**/**
Fluorescence	SiQDs	970	0.77–385 × 10^3^	20	0.997	[12]	Serum
Fluorescence	SQDs	262.41	0.5–40 × 10^3^	60	0.991	[119]	Serum
Fluorescence	pSi	80	0.16–6.5 × 10^3^	10	0.984	[123]	**/**
Fluorescence	AuNPs	9.25 × 10^−2^	7.5 × 10^−2^–7.5 × 10^4^	120	**/**	[124]	Blood
Fluorescence	CdSe QDs/PCL	**/**	200–2400	10	0.998	[121]	**/**
Fluorescence	Paper−based Sensor	0.225	0.2–3.13	˂20	0.997	[99]	Blood
Fluorescence	Aptamer-Coated Magnetic Beads	1.51 × 10^−2^	9.25 × 10^−3^–9.25 × 10^2^	60	0.990	[125]	**/**
Fluorescence	MoS_2_ nanosheets	5.09	0–578.13	10	0.990	[122]	**/**
Fluorescence	Magnetic Beads	2.75 × 10^−2^	0.025–6.25	15	**/**	[126]	Blood
Electrochemistry	Glassy Carbon Electrode	0.21	0.55–275	60	0.991	[110]	**/**
Electrochemistry	Screen-printed Electrode	50	50–500	10	0.998	[106]	**/**
Electrochemistry	N−Mo_2_C/SPE	25	60–700	**/**	0.991	[107]	Plasma
Electrochemistry	MP−dsSPCE	50	3.13–25	˂20	0.990	[109]	Blood
Electrochemistry	rGO−2DBioFET	7.22 × 10^−5^	7.22 × 10^−4^–9.25 × 10^3^	**/**	0.990	[127]	Serum
Electrochemistry	mPPS	0.5	**/**	**/**	**/**	[108]	Serum
Colorimetry	Pullulan-Based Inks	13	0–225	5	**/**	[98]	Serum
Colorimetry	LFA	2.5	1.25–125	15	0.960	[128]	Serum
Colorimetry	mAb−functionalized Magnetic Beads	2.41 × 10^−2^	6.48 × 10^−2^–46.25	15	**/**	[129]	Blood
Colorimetry	Magnetic Beads	0.24	**/**	30	**/**	[130]	**/**
Colorimetry	Paper-based Sensor	0.39	**/**	˂20	**/**	[99]	Blood
Colorimetry	Aptamer-Coated Magnetic Beads	0.57	9.25 × 10^−3^–9.25 × 10^2^	60	0.990	[125]	**/**
Colorimetry	Magnetic Beads	0.03	0.1–6.25	20	**/**	[126]	Blood
Chemiluminescence	Magnetic Beads	5 × 10^−3^	0.01–6.25	1	**/**	[126]	Blood
Immunoassay	TiO	12.8	1–100	30	0.996	[131]	**/**
Immunoassay	Microfluidic Microplate	6.25 × 10^−3^	**/**	˂90	**/**	[43]	Serum
Immunoassay	SPGE	0.45	0–0.17	120	0.991	[115]	Serum

## Data Availability

Not applicable.
